# High Glucose and Lipopolysaccharide Prime NLRP3 Inflammasome via ROS/TXNIP Pathway in Mesangial Cells

**DOI:** 10.1155/2016/6973175

**Published:** 2016-01-05

**Authors:** Hong Feng, Junling Gu, Fang Gou, Wei Huang, Chenlin Gao, Guo Chen, Yang Long, Xueqin Zhou, Maojun Yang, Shuang Liu, Shishi Lü, Qiaoyan Luo, Yong Xu

**Affiliations:** ^1^Department of Endocrinology, Affiliated Hospital of Luzhou Medical College, Luzhou, Sichuan 646000, China; ^2^Department of Internal Medicine, Nan'an District People's Hospital, Chongqing 400060, China; ^3^Department of Endocrinology, The Fifth People's Hospital of Chongqing, Chongqing 400062, China

## Abstract

While inflammation is considered a central component in the development in diabetic nephropathy, the mechanism remains unclear. The NLRP3 inflammasome acts as both a sensor and a regulator of the inflammatory response. The NLRP3 inflammasome responds to exogenous and endogenous danger signals, resulting in cleavage of procaspase-1 and activation of cytokines IL-1*β*, IL-18, and IL-33, ultimately triggering an inflammatory cascade reaction. This study observed the expression of NLRP3 inflammasome signaling stimulated by high glucose, lipopolysaccharide, and reactive oxygen species (ROS) inhibitor N-acetyl-L-cysteine in glomerular mesangial cells, aiming to elucidate the mechanism by which the NLRP3 inflammasome signaling pathway may contribute to diabetic nephropathy. We found that the expression of thioredoxin-interacting protein (TXNIP), NLRP3, and IL-1*β* was observed by immunohistochemistry in vivo. Simultaneously, the mRNA and protein levels of TXNIP, NLRP3, procaspase-1, and IL-1*β* were significantly induced by high glucose concentration and lipopolysaccharide in a dose-dependent and time-dependent manner in vitro. This induction by both high glucose and lipopolysaccharide was significantly inhibited by N-acetyl-L-cysteine. Our results firstly reveal that high glucose and lipopolysaccharide activate ROS/TXNIP/ NLRP3/IL-1*β* inflammasome signaling in glomerular mesangial cells, suggesting a mechanism by which inflammation may contribute to the development of diabetic nephropathy.

## 1. Introduction

Diabetic nephropathy (DN) is one type of microvascular complication of diabetes and the leading cause of end-stage renal disease in the Western world [1]. A primary hallmark of DN is the progressive damage and death of glomerular podocytes, resulting in renal sclerosis and fibrosis and the leaking of proteins into the urine. The onset of diabetic nephropathy is insidious and while the mechanism remains unclear, it is widely accepted that inflammation plays an important role [2]. The disorder of renal hemodynamics and metabolism caused by chronic hyperglycemia as well as hyperlipidemia may stimulate the secretion of inflammatory factors, leading to infiltration of immune cells in early diabetic nephrosis. Such immune-mediated inflammation is the essence of the microinflammatory state associated with innate immunity [3].

The mammalian immune system consists of two different arms: innate and adaptive immunity. Innate immunity is an evolutionarily conserved system that provides the first line of protection against invading microbes [4]. The NLRP3 (nucleotide-binding domain and leucine-rich repeat-containing family, pyrin domain-containing-3) inflammasome (also known as the NALP3 inflammasome) is an important component of the innate immune system and is composed of NLRP3, ASC (apoptosis-associated speck-like protein containing a CARD), and procaspase-1 [5]. The NLRP3 inflammasome senses endogenous and exogenous danger signals, such as lipopolysaccharide (LPS) and high glucose (HG), resulting in the activation of caspase-1 followed by activation of cytokines interleukin- (IL-) 1*β*, IL-18, and IL-33. This cascade triggers sustained inflammation, which has been associated with metabolic diseases [6, 7]. However, the role of the NLRP3 inflammasome in diabetic nephropathy remains unclear.

Lipopolysaccharide (LPS), often referred to as endotoxin, is found on the cell wall of Gram-negative bacteria. A high-fat diet increases the proportion of Gram-negative bacteria in the gut and causes a dramatic rise in the circulating concentration of plasma LPS, resulting in metabolic endotoxemia. Metabolic endotoxemia appears to be associated with a host of conditions including inflammation, obesity, type 2 diabetes, and metabolic syndrome [8, 9].

Thioredoxin-interacting protein (TXNIP) is an early response gene highly induced by diabetes and hyperglycemia [10–12]. TXNIP was initially identified as one of the proteins that interacts with thioredoxin (TRX) and reduces its function which scavenges reactive oxygen species (ROS) [13, 14]. Recent findings demonstrate a potential role for TXNIP in innate immunity via the NLRP3 inflammasome activation and release of IL-1*β* in diabetes and oxidative stress [15, 16]. Meanwhile, it is well known that oxidative stress plays an important role in the pathophysiology of diabetic nephropathy. However, a beneficial role of N-acetylcysteine (NAC) supplementation is discovered in oxidative stress [17]. NAC is an antioxidant that acts as a free radical scavenger [18]; it also activates glutathione (GSH), which acts intra- and extracellularly as an antioxidant eliminating ROS [19]. Based on these findings, we favor a model that ROS activate the NLRP3 inflammasome through the dissociation of TRX and TXNIP, contributing to the progression of diabetic complications [20, 21]. Therefore, we detected the expression of TXNIP, NLRP3, procaspase-1, and IL-1*β* in glomerular mesangial cells of diabetic nephropathy and then observed the changes of the players using NAC blocked ROS that stimulated by high glucose and LPS, aiming to elucidate the role of oxidative stress and the NLRP3 inflammasome signaling pathway in glomerular mesangial cells of diabetic nephropathy.

## 2. Materials and Methods

### 2.1. Establishing the Animal Model

Male Wistar rats weighing 200 g were purchased from the Biotechnology Corporation of Teng Xing (Chongqing, China). The rats were randomly allocated into two groups: a control group (NC group, *n* = 20) and a diabetic control group (DC group, *n* = 20). Rats in the diabetic control group were rendered diabetic by intraperitoneal injection of Streptozocin (STZ, Sigma, USA), at a dose of 60 mg/kg. STZ was dissolved in 0.1 M citrate buffer at pH4.5. Meanwhile, rats in the NC group received an intraperitoneal injection of the same volume of citrate buffer. After 3 days following the STZ injection, fasting glycemic measurements were performed in blood samples from tail veins, and blood glucose levels of *⩾*16.7 mmol/L lasting 3 days were confirmed as being “diabetic.”

### 2.2. Sample Collection

All rats were weighed and 24-hour urinary microalbumin was collected every day. After 6 or 8 weeks, all rats were sacrificed and heart blood was collected to measure BUN levels and fasting blood glucose (FBG) levels, using an automatic biochemistry analyzer. Both kidneys were cut along the coronal plane; upper poles of the right kidneys were used for pathology, and the left renal tissues were preserved at −80°C until required for Western blot analysis and RT-PCR.

### 2.3. Immunohistochemistry

Sections were incubated with the following primary antibodies: TXNIP (rabbit; 1 : 200; Abcam), NLRP3 (rabbit; 1 : 500; Abcam), and IL-1*β* (rabbit; 1 : 200; Abcam) over night at 4°C. After sections were washed with PBS, they were incubated with horseradish peroxidase-conjugated secondary antibodies (1 : 200 dilution) for 2 h at room temperature. For visualizing the signals, sections were treated with peroxidase substrate DAB (3, 3-diaminobenzidine) and counterstained with hematoxylin.

### 2.4. Renal Mesangial Cell Culture

Rat glomerular mesangial cells were cultured in Dulbecco's Modified Eagle Medium (DMEM) containing 5.6 mmol/L glucose and 10% fetal bovine serum (FBS) at 37°C and 5% CO_2_. The glomerular mesangial cells were used for all experiments and were randomly divided into the following six groups: (1) normal control group (group NC, 5.6 mmol/L glucose); (2) high glucose group (group HG, 10, 20, or 30 mmol/L glucose); (3) osmotic pressure group (group OP, 5.6 mmol/L glucose + 24.4 mmol/L mannitol); (4) NAC intervention in high glucose group (group HG + NAC, 10 *μ*mol/L NAC in 30 mmol/L glucose medium); (5) LPS group (group LPS, 1, 5, or 10 *μ*g/L LPS); and (6) NAC intervention in LPS group (group LPS + NAC, 10 *μ*mol/L NAC in 10 *μ*g/L LPS medium). The cells in each group were induced for 6, 12, or 24 h before NLRP3, procaspase-1, IL-1*β*, TXNIP protein, and mRNA levels were measured.

### 2.5. Western Blotting

Total protein was extracted from glomerular mesangial cells using a protein extraction kit (Kaiji, Shanghai, China). Proteins were separated by sodium dodecyl sulfate-polyacrylamide gel electrophoresis and transferred to a polyvinylidene difluoride (PVDF) membrane (Millipore). Immunoblotting was performed using anti-NLRP3 antibody (rabbit; 1 : 4,000; Abcam), anti-TXNIP antibody (rabbit; 1 : 800; Abcam), anti-procaspase-1 antibody (rabbit; 1 : 700; Cell Signaling Technology), and anti-*β*-actin antibody (mouse; 1 : 3,000; Beyotime).

### 2.6. Reverse-Transcription Polymerase Chain Reaction (RT-PCR)

Total RNA was isolated from glomerular mesangial cells using an RNA extraction kit (Tiangen Biotech, Beijing, China). Total RNA was reverse-transcribed (RT) using a Takara RNA PCR kit (Baoshengwu, Dalian, China). cDNA was amplified in a gradient thermal cycler (Eppendorf, Germany) using polymerase chain reaction (PCR) Master Mix (Baoshengwu, Dalian, China). Gene expression was normalized to *β*-actin. The primer sequences were as follows: NLRP3 (forward, 5′-CCA GGG CTC TGT TCA TTG -3′; reverse, 5′-CCT TCA CGT CTC GGT TC -3′), TXNIP (forward, 5′-CCA CGC TGA CTT TGA GAA CA -3′; reverse, 5′-GGA GCC AGG GAC ACT AAC ATA-3′), IL-1*β* (forward, 5′- CTT CAA ATC TCA CAG CAG CAT-3′; reverse, 5′- CAG GTC GTC ATC ATC CCA C-3′), and *β*-actin (forward, 5′-CGT TGA CAT CCG TAA AGA C-3′; reverse, 5′-TGG AAG GTG GAC AGT GAG -3′).

### 2.7. Data Analysis

All data obtained from at least three independent experiments were expressed as the mean ± standard deviation (SD) and analyzed using one-way analysis of variance (ANOVA), followed by the LSD post hoc test for multiple comparisons (SPSS 11.5 statistical software). *p* < 0.05 was considered significant.

## 3. Results

### 3.1. STZ-Induced Changes in 24-Hour Urine Protein and Renal Function of Diabetic Rats

Compared to the NC group, fasting blood glucose (FBG) levels, 24-hour urine protein, urinary albumin-to-creatinine ratios (ACR), and BUN levels were increased in the DC group.

### 3.2. The Expression of TXNIP, NLRP3, and IL-1*β* Was Observed In Vivo

**Figure 1:**
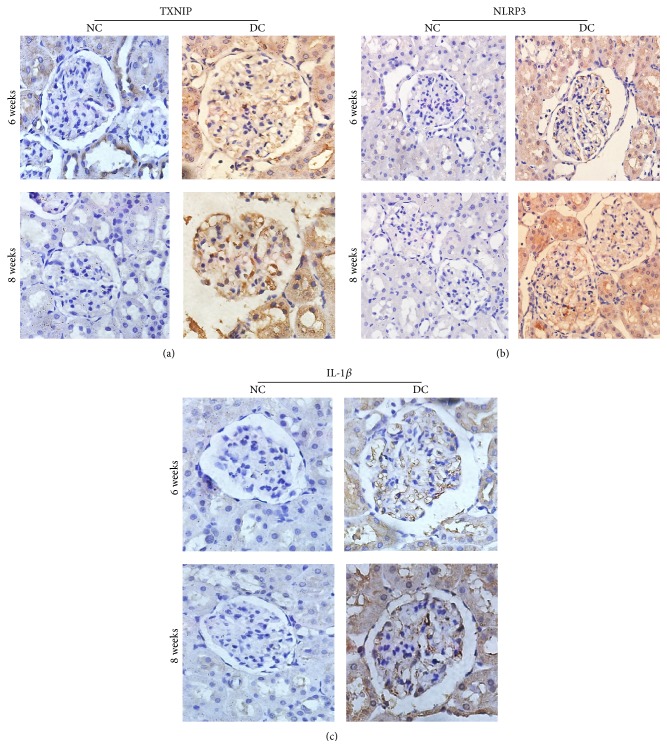
In the immunohistochemistry (×400 double), TXNIP, NLRP3, and IL-1*β* expression (a, b, and c) in the DC group were increased compared to the NC group.

Renal tissue immunohistochemistry (Figures 1(a), 1(b), and 1(c)) showed that, compared with the NC group, there were increased* TXNIP*,* NLRP3*,* and IL-1β* expressions that were particularly evident in the DC group. The expression of those players in 8 weeks was stronger than that in 6 weeks.

### 3.3. TXNIP, NLRP3, Procaspase-1, and IL-1*β* Expression Is Induced by High Concentrations of Glucose

**Figure 2:**
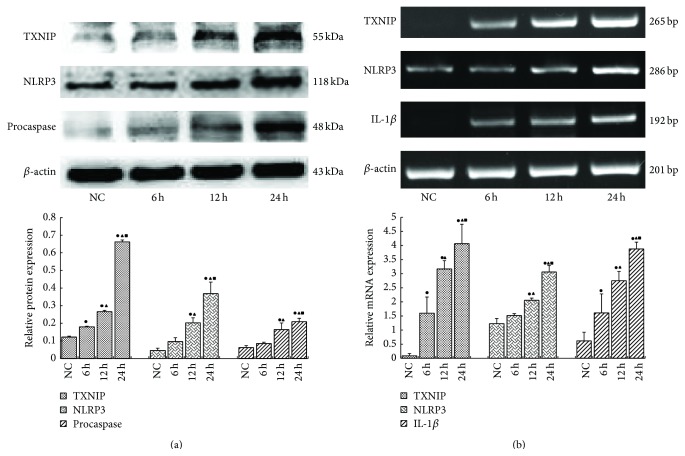
Compared to the normal control group, TXNIP, NLRP3, procaspase-1, and IL-1*β* were significantly induced at both the mRNA and protein levels following 6, 12, and 24 h of exposure to 30 mmol/L glucose. Moreover, protein and mRNA levels were highest at 24** **h (a, b) (^●^ *p* < 0.05 versus NC group, ^▲^ *p* < 0.05 versus 6 h, and ^■^ *p* < 0.05 versus 12 h).

Compared to the normal control group, TXNIP, NLRP3, procaspase-1, and IL-1*β* were significantly induced at both the mRNA and protein levels following 6, 12, and 24 h of exposure to 30 mmol/L glucose (*p* < 0.05). Moreover, protein and mRNA levels were highest at 24 h (Figures 2(a) and 2(b)), suggesting that a high glucose concentration increased NLRP3 inflammasome levels in a time-dependent manner.

**Figure 3:**
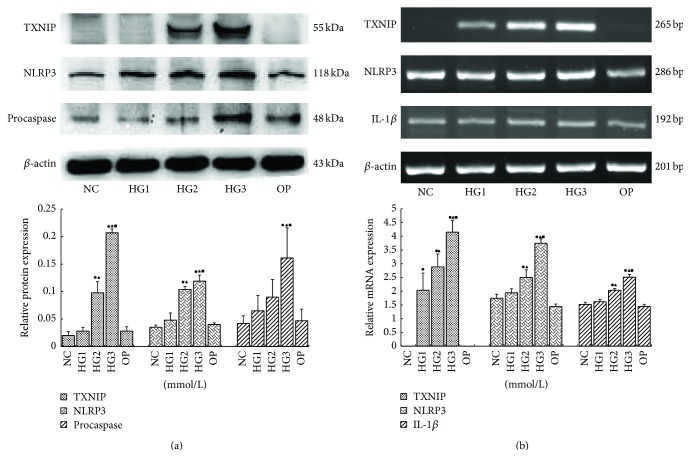
TXNIP, NLRP3, procaspase-1, and IL-1*β* were also significantly induced by several high concentrations of glucose at 24** **h. The highest relative expression of these factors was observed in the 30 mmol/L high glucose group (a, b) (NC: 5.6 mmol/L glucose; HG1: 10 mmol/L glucose; HG2: 20 mmol/L glucose; HG3: 30 mmol/L glucose; OP: 5.6 mmol/L glucose + 24.4 mmol/L mannitol) (^●^ *p* < 0.05 versus NC group or OP group, ^▲^ *p* < 0.05 versus HG1 group, and ^■^ *p* < 0.05 versus HG2 group).

TXNIP, NLRP3, procaspase-1, and IL-1*β* were also significantly induced by several high concentrations of glucose at 24 h (*p* < 0.05). The highest relative expression of these factors was observed in the 30 mmol/L high glucose group (Figures 3(a) and 3(b)), suggesting that glucose increased NLRP3 inflammasome levels in a dose-dependent manner. No genes were significantly induced in the osmotic pressure group (*p* > 0.05).

### 3.4. TXNIP, NLRP3, Procaspase-1, and IL-1*β* Expression Is Induced by LPS

**Figure 4:**
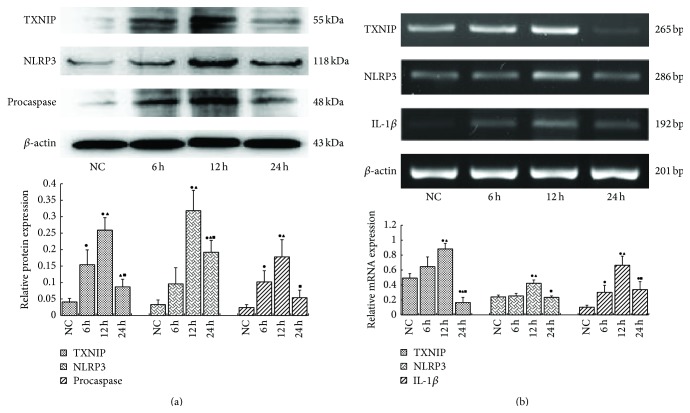
Compared to the normal control group, TXNIP, NLRP3, procaspase-1, and IL-1*β* were significantly induced at both the mRNA and protein levels following 6, 12, and 24 h of exposure to 10 *μ*g/L LPS (*p* < 0.05). Protein and mRNA levels were highest at 12 h (a, b) (^●^ *p* < 0.05 versus NC group, ^▲^ *p* < 0.05 versus 6 h, and ^■^ *p* < 0.05 versus 12 h).

Compared to the normal control group, TXNIP, NLRP3, procaspase-1, and IL-1*β* were significantly induced at both the mRNA and protein levels following 6, 12, and 24 h of exposure to 10 *μ*g/L LPS (*p* < 0.05). Protein and mRNA levels were highest at 12 h (Figures 4(a) and 4(b)).

**Figure 5:**
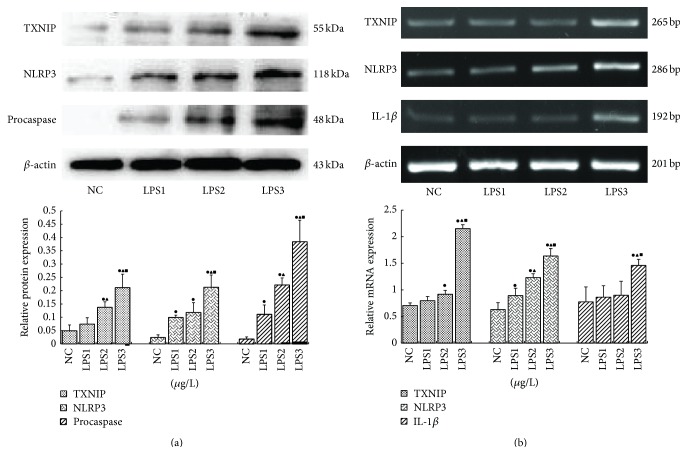
TXNIP, NLRP3, procaspase-1, and IL-1*β* were also significantly induced by different concentrations of LPS at 12 h. The highest relative expression of these factors was observed in the 10 *μ*g/L LPS group (a, b) (NC: 0 *μ*g/L LPS; LPS1: 1 *μ*g/L; LPS2: 5 *μ*g/L; LPS3: 10 *μ*g/L) (^●^ *p* < 0.05 versus NC group, ^▲^ *p* < 0.05 versus LPS1 group, and ^■^ *p* < 0.05 versus LPS2 group).

TXNIP, NLRP3, procaspase-1, and IL-1*β* were also significantly induced by different concentrations of LPS at 12 h (*p* < 0.05). The highest relative expression of these factors was observed in the 10 *μ*g/L LPS group (Figures 5(a) and 5(b)), suggesting that LPS increased NLRP3 inflammasome levels in a dose-dependent manner.

### 3.5. Induction of TXNIP, NLRP3, Procaspase-1, and IL-1*β* Is Inhibited by NAC

**Figure 6:**
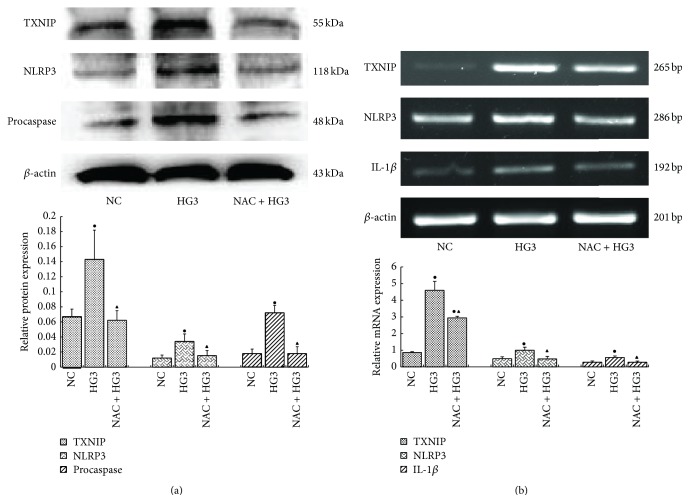
Compared with the high glucose group (30 mmol/L), mRNA and protein levels of TXNIP, NLRP3, procaspase-1, and IL-1*β* were significantly lower in the high glucose (30 mmol/L) plus NAC group (a, b) (NC: 5.6 mmol/L glucose; HG3: 30 mmol/L glucose; NAC + HG3: 10 *μ*mol/L NAC + 30 mmol/L glucose) (^●^ *p* < 0.05 versus NC group, ^▲^ *p* < 0.05 versus HG3 group).

Compared with the high glucose group (30 mmol/L), mRNA and protein levels of TXNIP, NLRP3, procaspase-1, and IL-1*β* were significantly lower in the high glucose (30 mmol/L) plus NAC group (*p* < 0.05) (Figures 6(a) and 6(b)), suggesting that NAC inhibited induction by glucose.

**Figure 7:**
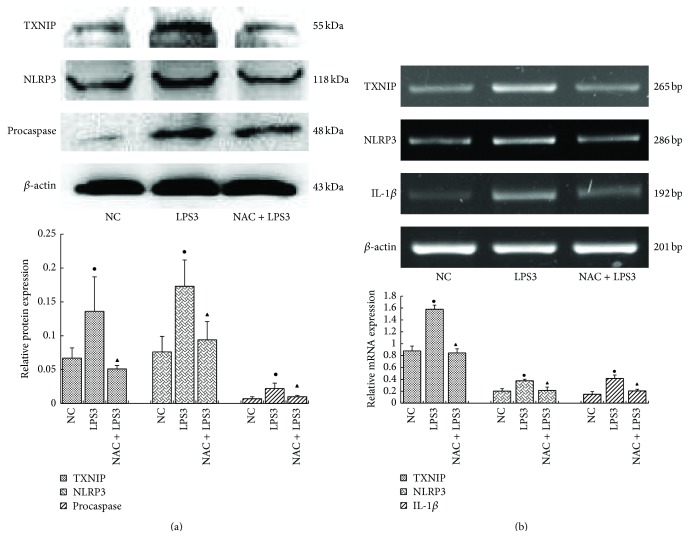
Compared with the LPS (10 *μ*g/L) group, mRNA and protein levels of TXNIP, NLRP3, procaspase-1, and IL-1*β* were significantly lower in the LPS (10 *μ*g/L) plus NAC group (a, b) (NC: 0 *μ*g/L LPS; LPS3: 10 *μ*g/L LPS; NAC + LPS3: 10 *μ*mol/L NAC + 10 *μ*g/L LPS) (^●^ *p* < 0.05, versus NC group, ^▲^ *p* < 0.05 versus LPS3 group).

Compared with the LPS (10 *μ*g/L) group, mRNA and protein levels of TXNIP, NLRP3, procaspase-1, and IL-1*β* were significantly lower in the LPS (10 *μ*g/L) plus NAC group (*p* < 0.05) (Figures 7(a) and 7(b)), suggesting that NAC inhibited induction by LPS.

## 4. Discussion

Diabetic nephropathy is characterized by chronic low-grade inflammation due to infiltration of immune cells and cytokines in kidney tissues. The immune-mediated inflammatory response is a key component of hyperglycemia and diabetic nephropathy. Inflammatory cytokines such as IL-1*β*, IL-18, TNF-*α*, MCP-1, and ICAM-1 are significantly increased in renal tissues during diabetic nephropathy and attenuating the expression of these cytokines may protect against diabetic renal injury [22].

IL-1*β* is not only a key contributor to obesity-induced inflammation and subsequent insulin resistance but also type 2 diabetes [6]. The level of IL-1*β* gradually increases from the development of normal glucose tolerance to impaired glucose tolerance to type 2 diabetes and is positively correlated with insulin resistance. Furthermore, IL-1*β* is also an instigator of the inflammation found in diabetic nephropathy. Hyperglycemia stimulates the secretion of macrophage-produced cytokine IL-1*β*, which induces inflammatory cytokine production and invasion into renal tissues [23]. Recent randomized clinical trials demonstrated that the level of IL-1*β* level was significantly increased in and positively correlated with renal function injury in patients with diabetic nephropathy [24, 25]. Here we showed that the IL-1*β* was significantly upregulated in the early stage of diabetic nephropathy, and the expression of IL-1*β* in glomerular mesangial cells was induced by high glucose and lipopolysaccharide in a dose-dependent and time-dependent manner. These results suggest that high glucose and lipopolysaccharide induce a renal inflammatory reaction and IL-1*β* contributes to the pathogenesis of diabetic nephropathy, supporting the role of microinflammation in diabetic nephropathy.

The secretion of bioactive IL-1*β* is predominantly controlled by activation of caspase-1 through the assembly of a multiprotein scaffold inflammasome composed of NLRP3, ASC, and procaspase-1 [26]. NLRP3 is mainly responsible for the detection of infection-derived molecules such as lipopolysaccharide. Moreover, NLRP3 is unique in its ability to recognize molecular patterns associated with host-derived signals that are abundant in obese individuals, including excess ATP, glucose, ROS, and uric acid, as well as crystals of cholesterol [5]. ASC is the indispensable adaptor that connects NLRP3 and procaspase-1 [27]. Caspase-1 mediated cleavage is the limiting step for processing IL-1*β* into its secreted active forms. Inactive procaspase-1 molecules are recruited to the NLRP3 inflammasome [28]. The NLRP3 inflammasome is not only an important sensor of metabolic dysregulation, but also a molecular platform of procaspase-1 and IL-1*β* activation [26].

Mechanisms leading to NLRP3 inflammasome activation are a matter of debate. Several models are widely favored in the literature, including the K^+^ channel model, lysosomal damage model, and ROS model, although they may not be mutually exclusive [16, 29, 30]. All NLRP3 agonists trigger the production of ROS, which leads to the activation of the NLRP3 inflammasome via the ROS-sensitive TXNIP protein [31].

However, the role and mechanism of NLRP3 inflammasome in diabetic nephropathy is not yet fully understood. Chen and colleagues discovered that ATP-P2X signaling mediates high glucose-induced activation of the NLRP3 inflammasome and IL-1 family cytokine secretion, causing the development of inflammation in renal tubular epithelial cells [24]. However, our results firstly demonstrate the activation of NLRP3 inflammasome following high glucose-induced expression of NLRP3 and procaspase-1 in mesangial cells in vivo and vitro. Taken together, these results reveal that high glucose activates the NLRP3 inflammasome and mediates inflammation in diabetic nephropathy. In addition, sustained hyperglycemia may increase uric acid and fatty acid levels in circulation. Hyperglycemia along with uric acid and fatty acid activates the NLRP3 inflammasome, which is involved in the occurrence and development of inflammation in diabetic nephropathy [32–34].

Endotoxin is associated with an increased risk for diabetes. Importantly, the risk is independent of other established risk factors like glucose and lipid levels [35]. Additionally, several clinical trials provide evidence that circulating lipopolysaccharide is higher in diabetic patients with kidney disease compared with nondiabetic nephropathy patients, and metabolic endotoxemia is also associated with the development of diabetic nephropathy [36, 37]. Endotoxemia activates the innate immune system, characterized by a release of antibodies, cytokines, and other inflammatory mediators, which may promote kidney injury [38]. Our experimental results showed consistently increased expression of NLRP3 and procaspase-1 in mesangial cells treated with lipopolysaccharide. Thus, during metabolic endotoxemia, a large amount of lipopolysaccharide from the intestine that goes into the blood may activate the renal NLRP3 inflammasome, resulting in the release of IL-1*β* and promotion of kidney damage [39].

Oxidative stress created by hyperglycemia plays an important role in the pathology of diabetic nephropathy. Thioredoxin was initially identified as a protein that scavenges ROS and maintains cellular activity [40]. TXNIP is an endogenous inhibitor that interacts with thioredoxin, reducing its function to mediate oxidative stress. TXNIP is significantly increased in rats and humans with diabetic nephropathy and closely correlated with urinary albumin, renal fibrosis, and reactive oxygen species [41, 42]. We found that TXNIP was significantly induced by high glucose and LPS, even at 6 h, in our in vitro study. Our results suggest that TXNIP is an early response gene that is highly induced by hyperglycemia and diabetic nephropathy. Well, the expression of TXNIP, NLRP3, procaspase-1, and IL-1*β* was significantly increased by high glucose concentration and LPS in a dose-dependent and time-dependent manner in vitro. It indicated a subtle relationship between the players. Surprisingly, the induction of TXNIP, NLRP3, procaspase-1, and IL-1*β* by both high glucose and LPS was significantly inhibited by NAC intervention. It reveals a close connection between the players and an activation of ROS in the TXNIP pathway. ROS promotes the activation of TXNIP pathway; however, scavenging ROS may inhibit the activation of it. Additionally, in our previous studies, pathological changes in the kidney were obvious, followed by the upregulation of the proposed players in diabetic rats; the glomerular tuft and mesangial area were increased by HE staining [43]. There was a trend for an increase of glomerular volume in diabetic rats compared with normal rats. Collagen plays a critical structural role in renal fibrosis of DN. Observation with the light microscope, following Masson staining, demonstrated that accumulation of collagen in the kidney of the diabetic rats was greater than the normal rats in gross appearance [43]. These experiments and figures indicated that the proposed proteins in TXNIP pathway may play an important role in the inflammation of incipient diabetic nephropathy, renal sclerosis, and fibrosis. Based on our findings, we suggest a new model for the activation of the NLRP3 inflammasome and development of diabetic neuropathy. Exposure of the kidneys to high glucose and lipopolysaccharide results in the production of a massive amount of reactive oxygen species, which causes the TXNIP bound to thioredoxin to disassociate. TXNIP reduces the ROS scavenging capacity of thioredoxin and binds to NLRP3, mediating NLRP3 inflammasome assembly with ASC and procaspase-1. The subsequent autocleavage and activation of caspase-1 in turn results in the processing of pro-IL-1*β* to its mature form, which then leads to the induction of other proinflammatory genes, eventually promoting the oxidative stress and inflammation present in diabetic nephropathy [20, 44].

Although current treatments which concentrate on controlling hyperglycemia and hypertension reduce the risk of progressive kidney disease, diabetic kidney disease remains the leading cause of ESRD and the major risk amplifier for death in the population [45]. Therefore, novel therapeutic approaches are urgently needed, while a growing body of evidence from human, animal, and in vitro studies indicates that existing drugs, including the urate-lowering agent allopurinol, the anti-TNF agents etanercept, endothelin antagonist avosentan, and the immunomodulating drug abatacept, might be effective in preventing or slowing the progression of diabetic nephropathy to end-stage renal disease by targeting metabolic, inflammatory, and immunological pathways [45–47]. Rodrigues and colleagues showed that P2X7 receptor, which is also related to oxidative stress and induces tissue apoptosis or necrosis, was inhibited in diabetic rats treated with NAC [48]. They suggest that the maintenance of redox homeostasis could be useful as coadjuvant treatment to delay the progression of diabetic nephropathy. The P2 purinergic receptors, such as P2X7 receptor, modulate a variety of physiologic events upon the maintenance of a highly sensitive “set point,” the derangement of which may lead to the development of key pathogenic mechanisms during acute and chronic diseases. Solini and colleagues suggest that extracellular ATP signaling via P2 purinergic receptors may be involved in different renal pathologic conditions [49]. This review summarizes that NAC is potential therapeutic options targeting due to inhibiting the activation of TXNIP signal and extracellular ATP signaling. Nevertheless, there is a contradiction that NAC in moderate doses given over a month did not have significant effect on the overall oxidative stress in patients with DN and did not reduce proteinuria [50]. It shows that reactive oxygen species are not the only signal to induce oxidative stress in vivo. There must be else mechanisms such as inflammation, polyol pathway, and advanced glycation end products, involving in oxidative stress. Therefore, NAC do not completely inhibit the activation of oxidative stress. Moreover, NAC did not achieve the expected results in patients with DN, maybe due to not enough treatment time, not powerful reduction of oxidative stress, and so on.

## 5. Conclusion

Our study has firstly demonstrated that high glucose and lipopolysaccharide can activate the pathway of ROS/TXNIP/NLRP3 inflammasome signaling and results in the release of IL-1*β* in glomerular mesangial cells. These results help to clarify the cellular and molecular basis of the association between innate immunity and diabetic nephropathy, suggesting a new target for treatment of diabetic nephropathy. Future studies will focus on the interaction among pathogen-associated molecular patterns, damage-associated molecular patterns, and innate immunity in order to clarify the molecular mechanisms behind the development of metabolic diseases. Such findings have the potential which profoundly impact the prevention of diabetes and associated complications.
